# Exploiting *in silico* structural analysis to introduce emerging genotype–phenotype correlations in DHCR24-related sterol biosynthesis disorder: a case study

**DOI:** 10.3389/fgene.2023.1307934

**Published:** 2024-01-04

**Authors:** Dario Cocciadiferro, Tommaso Mazza, Davide Vecchio, Tommaso Biagini, Francesco Petrizzelli, Emanuele Agolini, Andrea Villani, Daniele Minervino, Diego Martinelli, Cristiano Rizzo, Sara Boenzi, Filippo Maria Panfili, Paola Sabrina Buonuomo, Marina Macchiaiolo, Andrea Bartuli, Antonio Novelli

**Affiliations:** ^1^ Translational Cytogenomics Research Unit, Bambino Gesù Children’s Hospital, IRCCS, Rome, Italy; ^2^ Bioinformatics Unit, Fondazione IRCCS Casa Sollievo Della Sofferenza, San Giovanni Rotondo, Italy; ^3^ Rare Diseases and Medical Genetics Unit, Bambino Gesù Children’s Hospital, IRCCS, Rome, Italy; ^4^ Division of Metabolic Diseases, Bambino Gesù Children’s Hospital IRCCS, Rome, Italy

**Keywords:** DHCR24, desmosterolosis, genotype–phenotype correlation, structural biology, medical genetics, bioinformatics

## Abstract

Desmosterolosis is a rare sterol biosynthesis disorder characterized by multiple congenital anomalies, failure to thrive, severe developmental delay, progressive epileptic encephalopathy, and elevated levels of desmosterol caused by biallelic mutations of *DHCR24* encoding 3-β-hydroxysterol Δ-24-reductase. DHCR24 is regarded as the key enzyme of cholesterol synthesis in the metabolism of brain cholesterol as it catalyzes the reduction of the Δ-24 double bond of sterol intermediates during cholesterol biosynthesis. To date, 15 *DHCR24* variants, detected in 2 related and 14 unrelated patients, have been associated with the desmosterolosis disorder. Here, we describe a proband harboring the never-described *DHCR24* homozygous missense variant NM_014762.4:c.506T>C, NP_055577.1:p.M169T, whose functional validation was confirmed through biochemical assay. By using molecular dynamics simulation techniques, we investigated the impact of this variant on the protein stability and interaction network with the flavin adenine dinucleotide cofactor, thereby providing a preliminary assessment of its mechanistic role in comparison to all known pathogenic variants, the wild-type protein, and a known benign *DHCR24* variant. This report expands the clinical and molecular spectra of the DHCR24-related disorder, reports on a novel DHCR24 deleterious variant associated with desmosterolosis, and gives new insights into genotype–phenotype correlations.

## 1 Introduction

Desmosterolosis (MIM 602398) is a rare autosomal recessive disorder of cholesterol biosynthesis characterized by the accumulation of desmosterol in plasma and tissues, leading to a wide range of clinical features including intellectual disability, developmental delay, epilepsy, cataracts, skeletal abnormalities, and other congenital malformations. Common brain abnormalities associated with desmosterolosis include malformation of the corpus callosum and loss of white matter. Individuals with desmosterolosis often experience spasticity and rigid joints in their hands and feet, along with other features such as abnormal head size, short stature, micrognathia, cleft palate, nystagmus or strabismus, heart defects, and seizures (Horvat et al., 2011; Zolotushko et al., 2011; Rohanizadegan and Sacharow, 2018). Patients with desmosterolosis carry high levels of cholesterol precursor desmosterol in plasma, tissue, and cultured cells, caused by the deficiency of the enzyme 3β-hydroxysterol Δ24-reductase (DHCR24), which catalyzes the reduction of the Δ24 double bond of sterol intermediates in cholesterol biosynthesis. As a member of the flavin adenine dinucleotide (FAD)-dependent oxidoreductases, DHCR24 requires the flavin adenine dinucleotide (FAD) for cholesterol catalysis. Indeed, the conversion of desmosterol to cholesterol by DHCR24 *in vitro* is strictly dependent on reduced nicotinamide adenine dinucleotide phosphate and is two-fold increased by the addition of FAD to the assay (Waterham et al., 2001). DHCR24 is involved in multiple cellular functions; in particular, it modulates lipid raft formation, thus facilitating signal transduction and trafficking. It is also involved in regulating oxidative stress and has neuroprotective, anti-apoptotic, and anti-inflammatory properties (Horvat et al., 2011).

*DHCR24* variants have been associated with desmosterolosis, resulting in the production of altered 24-dehydrocholesterol reductase and consequent decreased cholesterol production. As a result, brain cells, which rely entirely on cellular cholesterol production, are severely affected. Without adequate cholesterol, the proper formation of cell membranes is disrupted, and nerve cells are not protected by myelin, leading to cell death. The reduction in cholesterol production also has more severe effects before birth because of the rapid increase in cell number that occurs during this period. This disruption in normal cell formation before birth is likely responsible for the additional developmental abnormalities associated with desmosterolosis (Porter and Herman, 2011). Indeed, previous studies showed that DHCR24 has a remarkable decline in the brain of Alzheimer disease (AD) patients and might be involved in other pathological injuries that are related to AD, such as autophagy, mitochondrial injuries, inflammation, neurosteroid synthesis, and other metabolic abnormalities (Greeve et al., 2000; Lu et al., 2014; Bai et al., 2021; Bai et al., 2022). To date, 15 *DHCR24* variants, from 2 related and 14 unrelated patients, have been identified in association with desmosterolosis with a variable degree of neurological impairment; of them, 11 were missense variants in homozygosity or compound heterozygosity, and 2 were nonsense variants with either 1 frameshift or 1 splicing variant in the compound heterozygous state; all were classified as pathogenic or likely pathogenic in HGMD release 2022.1 (Stenson et al., 2020).

Here, we describe a new patient with severe developmental delay, epileptic encephalopathy, spastic tetraparesis, cataracts, and several dysmorphic features, harboring the novel homozygous missense variant NM_014762.4:c.506T>C, NP_055577.1:p.M169T in the *DHCR24* gene. By using molecular dynamics simulation techniques, we first investigated the impact of this variant on the protein stability and the residue interaction network with FAD. Then, we assessed its mechanistic consequences on the protein structure and functions in comparison with all known pathogenic variants, a further benign variant, and the wild-type protein. Finally, we derived distinct molecular aspects of the considered mutant proteins which were all associated with the disease manifestation.

## 2 Methods

### 2.1 Data collection

Phenotypes associated with *DHCR24* variants causing desmosterolosis based on the literature review are reported in Supplementary Table S3.

### 2.2 Whole-exome sequencing

Whole-exome sequencing (WES) was performed on the proband and parents’ DNA using the Twist Human Core Exome Kit (Twist Bioscience), according to the manufacturer’s protocol, and sequenced on the Illumina NovaSeq 6000 platform. The BaseSpace pipeline and Geneyx software (LifeMap Sciences) were, respectively, used for variant calling and annotation. Sequencing data were aligned to the hg19 human reference genome. Relatedness between parents was assessed using the KING toolset version 2.3.1. The potential pathogenic impact of the *DHCR24* variant NM_014762.4:c.506T>C, NP_055577.1:p.M169T was preliminarily predicted using 14 distinct software packages, namely, SIFT, PolyPhen2, LRT, MutationTaster, MutationAssessor, FATHMM, PROVEAN, MetaSVM, MetaLR, M-CAP, CADD, DANN, fathmm-MKL, and GenoCanyon. Evolutionary conservation was instead assessed using GERP++, phyloP, phastCons, and SiPhy. The global minor allele frequency (MAF) for the analyzed variants was obtained from the Genome Aggregation Database (gnomAD ver. 2.1.1). Based on the guidelines of the American College of Medical Genetics and Genomics (ACMG), a minimum depth coverage of 30× was considered suitable for analysis. Variants were visualized using the Integrative Genomics Viewer (IGV).

### 2.3 Biochemical analysis

The Tri-Sil HPP reagent was obtained from Thermo Scientific-USA; sterol standards and other HPLC-grade organic solvents and highest-purity chemical reagents were obtained from Merck KGaA, Darmstadt, Germany. Plasma desmosterol and other neutral sterols were analyzed by gas chromatography–mass spectrometry (GC/MS), according to the method described by Kelley (1995) with slight modifications (Kelley, 1995). In brief, 200 *μL* of plasma, 100 *μL* of the internal standard solution (5-α-cholestane 10 *μmol/L* in methanol), and 3 ml of 4% (w/v) KOH in 90% ethanol were placed into a 14 × 100-*mm* screw-cap culture tube. The tube was heated at 100°C for 1 h to hydrolyze the sample.

After hydrolysis, the sample was left to cool at room temperature and successively extracted three times with 2 ml of hexane by vigorous shaking (1 *min*). The mixture was centrifuged (3000 *rpm*, 5 *min*). The organic layers were combined in a 2-ml glass vial, evaporated under a nitrogen stream at 40°C, and derivatized with 100 *µL* of Tri-Sil HPP reagent at 60°C for 30 *min*. The analysis was performed by GC/MS (Agilent Technologies Model 7890A GC and Agilent Technologies Model 5975C MS). Then, 1 μ*L* of the derivative mixture was injected into the splitless injector port GC, leading to a 0.25 *mm* × 30-m Agilent J&W DB-5MS UI (0.25-*µm* film) capillary column. The injection port temperature was 250°C. After 1 min at 200°C, the oven temperature was raised to 280°C at 15°C/*min* and finally held at 280°C for 15 min before recycling. MS was operated in the EI mode at 70 *eV* with an ion source temperature of 280°C. MS acquisition modes were as follows: the full scan mode (from 50 to 550 *amu*) and selected-ion monitoring mode (SIM). SIM ions collected between 8 and 21 *min* were m/z = 357 and 372 for 5-a-cholestane; m/z = 329 and 368 for cholesterol; m/z = 445 and 460 for cholestanol; m/z = 325 and 351 for 7DHC and 8DHC, respectively; m/z = 343 and 351 for desmosterol; and m/z = 255 and 458 for lathosterol. Neutral sterol quantification was performed in the SIM mode by internal standard calibration.

### 2.4 Structural characterization

#### 2.4.1 Molecular modeling and system preparation

Atomic coordinates of the DHCR24 protein (UniProt ID = Q15392) were retrieved from the AlphaFold Protein Structure Database (Varadi et al., 2022), ensuring that the resulting modeled protein exhibited per-residue confidence scores (pLDDT) ranging from “Confident” to “Very high”. Relevant functional cofactors like FAD and relative binding pockets were first inferred using AlphaFill (Hekkelman et al., 2023) and then refined by minimization.

Then, the wild-type model was mutated *in silico* to introduce the following missense variants: p.M169T, p.E480K, p.R94H, p.R103C, p.L167S, p.E191K, p.Y471S, p.D473N, p.L324W, p.N294T, and p.K306N. The latter two variants were treated both individually and as double mutants. Finally, a putative benign variant, p.D508N (MAF: 0.002245 and reported as benign in ClinVar), was introduced as a control.

The web-based tool CHARMM-GUI Enhanced Sampler (Suh et al., 2022) was used to prepare both the wild-type and mutant systems and generate input files for the final molecular dynamics (MD) simulations. In detail, each system was embedded in a simulation box, extending up to 12 Å, filled with TIP3P water molecules, and Na^+^ and Cl^−^ counter ions were added to neutralize the overall charge of the system. Then, each model was subjected to energy minimization using steepest descent, followed by conjugate gradient methods, and gradually heated and equilibrated using a time step of 1 *fs* (Biagini et al., 2018; Biagini et al., 2019).

#### 2.4.2 Molecular dynamics simulation

MD simulation represents a powerful approach for examining the dynamic behavior of macromolecules as they move over time. However, the main limitation of classic MD computational approaches is represented by the simulation time scales, which are frequently insufficient for visualizing the event being studied. For this reason, enhanced MD approaches have been implemented. Here, we employed Gaussian accelerated molecular dynamics (GaMD) (Miao et al., 2015), an enhanced sampling method that adds a harmonic boost potential to smooth biomolecular potential energy surfaces and reduces energy barriers.

The implemented GaMD simulation protocol was already successfully tested in previous works (Petrizzelli et al., 2020; Biagini et al., 2022); in brief, it includes a preparatory ∼2 *ns* classic MD run to collect potential statistics and obtain the GaMD acceleration parameters; it is then followed by an 8-*ns* equilibration run. Finally, 200 *ns* of “dual-boost” GaMD simulations are carried out and divided into *n* sequential production steps. The simulations were performed using the Amber Molecular Dynamics package, version 20 (Case et al., 2005).

#### 2.4.3 Molecular dynamics analysis

GROMACS version 2018 was used to calculate the root-mean-square deviation (RMSD), measuring the average distance over time between C_α_ atoms compared to the starting minimized structure. Principal component analysis (PCA) was performed using gmx_covar, a tool implemented in GROMACS, to analyze the fluctuations of C_α_ atoms and identify patterns of collective motion at a larger scale, allowing the identification of a low-dimensional subspace that is likely to contain essential protein movements. In this way, the temporal dynamics of both the wild-type and mutant proteins were recreated using the 2D projections with respect to the first two eigenvectors obtained using the g_anaeig tool, while the conformational changes were investigated using a custom Python script that generates dynamic cross-correlation maps (DCCMs) starting from a covariance matrix.

Finally, GetContacts (https://getcontacts.github.io) was used to compute the fundamental interactions (i.e., hydrogen bonds, salt bridges, and van der Waals contacts) between DHCR24 and the FAD cofactor in every simulated time step. Images and 3D motions were generated using ChimeraX (Pettersen et al., 2021).

In order to cluster the DHCR24 dynamics of the mutant and wild-type proteins, we counted all the different fundamental protein–FAD interactions inferred by GetContacts. For each simulation and each interaction type, we retrieved the total number of time frames where an interaction occurred. Starting from these data, we performed a PCA transformation, followed by a Euclidean hierarchical clustering analysis, with the aim of comparing the MD simulations.

## 3 Results

### 3.1 Clinical description

**FIGURE 1:**
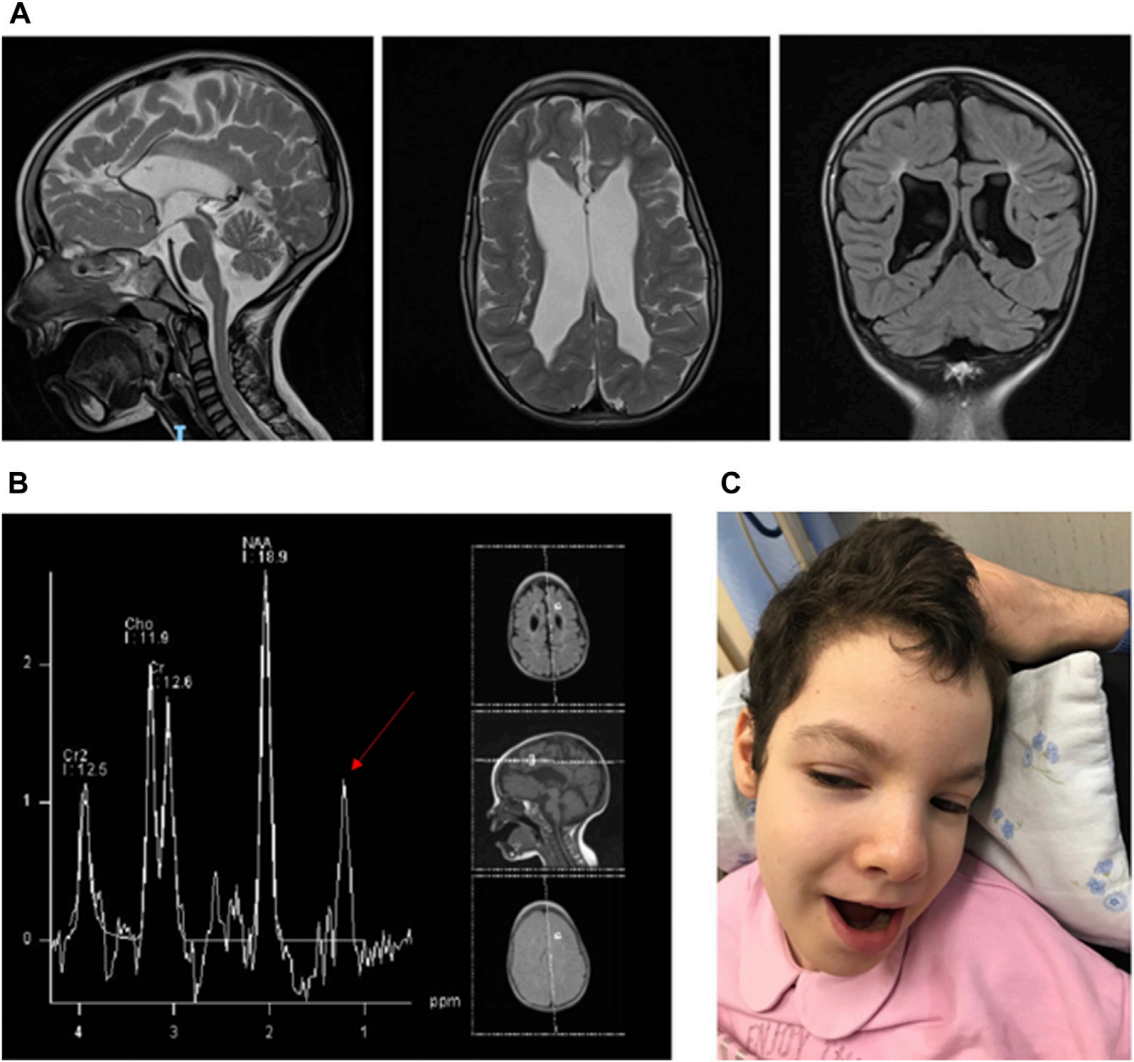
**(A)** Proband’s brain MRI showing cerebral and cerebellar atrophy with a simplified gyral pattern and conspicuous enlargement of the supratentorial ventricular system. **(B)** Proband’s brain MR spectroscopy showing abnormal lipid and choline concentration peaks (red arrow). **(C)** Proband’s facial features: coarse face, macrocephaly with metopic suture prominence and scapho-dolichocephalic appearance of the skull, thick eyelashes, slight bilateral epicanthus, bulbous nose tip, slightly anteverted nostrils with prominent columella, long philtrum, and a thin upper lip.

The proband is a female, fourth child of healthy parents coming from a small inland Italian town of approximately 8,000 inhabitants. She was born from the sixth out of seven pregnancies; the first characterized by fetal death at the eighth month of gestation likely due to unspecified fetal malformations and anhydramnios, and the fourth interrupted due to spontaneous miscarriage at the 10th week of gestation (WG). The parents’ family history was not informative for any genetic diseases. The parents declared no known consanguinity. From the morphological scan examination at 20 WG afterward, the proband’s pregnancy was characterized by the detection of bilateral ventriculomegaly and further confirmed through a fetal brain magnetic resonance imaging (MRI) scan. She was born at term by an elective cesarean section at 38,2 WG; at birth, her parameters were weight: 3,250 g (47th centile), head circumference: 36.5 *cm* (97th centile), and length: 50 *cm* (90th centile). Apgar scores were 9 at 1 min and 9 at 5 *min*. Her neonatal period was characterized by several episodes of bradycardia and desaturation associated with paroxysmal movements, marked feeding difficulties, lack of coordination in swallowing, and hypotonia. Hence, this neurophenotype was further investigated by EEG showing continuous and asymmetric slow temporal waves and through a novel brain MRI, which highlighted generalized parenchymal atrophy, reduced frontal and temporal opercularization and hypoplasia of the corpus callosum, basal ganglia, thalami, and spinal cord. Other findings were also depicted at the spectroscopy study such as N-acetylaspartate reduction with increased levels of myo-inositol and lactate. An antiepileptic therapy with phenobarbital was established, becoming ineffective at the age of 2 months when it was replaced with levetiracetam. The following clinical history was characterized by severe failure to thrive and developmental delay, progressive spastic quadriplegia, and visual impairment, due to diffused bilateral central pulverulent cataracts and retinal aberration mainly affecting the cones. Over time, the patient developed weekly fronto-central focal seizures and dystonic spasms. A novel EEG showed frontal epileptic anomalies. Hearing function, heart, and abdominal ultrasound scan tested normal. At the age of 8 years, she underwent a second brain MRI, showing a progression of cerebral and cerebellar atrophy with the simplified gyral pattern and conspicuous enlargement of the supratentorial ventricular system (Figure 1A). Moreover, a novel spectroscopic examination showed an abnormal lipid peak and choline concentration (Figure 1B). Thus, an extensive metabolic work-up was performed including plasmatic and urinary amino acids plus urinary organic acid testing, plasma lactate and pyruvate analyses, and determination of the pyruvic dehydrogenase complex activity on muscle homogenate and cultured fibroblasts, whose parameters tested negative and/or within the normal range.

The clinical picture was then further investigated by a standard karyotype and a CGH array analysis performed on DNA extracted from cultured amniocytes, respectively, resulting in a 46, XX normal female karyotype and arr (1–22,X)x2, which did not show any chromosomal aneuploidies and/or genomic cryptic rearrangements. The *GFAP* gene (associated with Alexander disease) Sanger sequence analysis and a next-generation sequencing panel of eight genes associated with pyruvate dehydrogenase complex defects (*DLAT*, *DLD*, *NFUI*, *PDHA1*, *PDHB*, *PDHX*, *PDK1*, and *PDK4* genes) did not show any deleterious variants.

The patient came to our observation at the age of 11 years. Auxological parameters were as follows: weight: 22 Kg (<3rd centile), head circumference: 51 cm (3rd centile), and length: 123 cm (<3rd centile). At the dysmorphological evaluation, several features were noted as follows: coarse facial features, relative macrocephaly with metopic suture prominence and scapho-dolichocephalic appearance of the skull, thick eyelashes, slight bilateral epicanthus with mild downslanting palpebral fissures, bulbous nose tip, slightly anteverted nostrils with prominent columella, long philtrum with a thin upper lip, and retrognathia (Figure 1C). At neurological evaluation, she showed severe spastic quadriplegia, multiplanar nystagmus, and bilateral exotropia.

### 3.2 Genetic and bioinformatic analyses

A WES analysis was performed on the proband and parents’ DNA. Relatedness between the parents was first assessed by analyzing the subject’s variants with the KING toolset and then excluded (kinship coefficient = −0.2366). WES revealed the homozygous missense variant NM_014762.4:c.506T>C, NP_055577.1:p.M169T in the *DHCR24* gene, inherited from the heterozygous carrier parents. This variant has never been reported; it is predicted as disease-causing by 12 software predictors out of 14, showing high congruency in the overall assessment (Castellana and Mazza, 2013) (Supplementary Table S1). Moreover, it exhibits an AlphaMissense pathogenicity score of 0.823 that further supports a likely pathogenic prediction. Coherently, it can also be classified as likely pathogenic based on the ACMG criteria including PM1, PM2, PP2, and PP3. Moreover, the variant resulted in being phylogenetically conserved through vertebrates and Mammalia by four distinct bioinformatics resources (Supplementary Table S1).

### 3.3 Biochemical analysis

**FIGURE 2:**
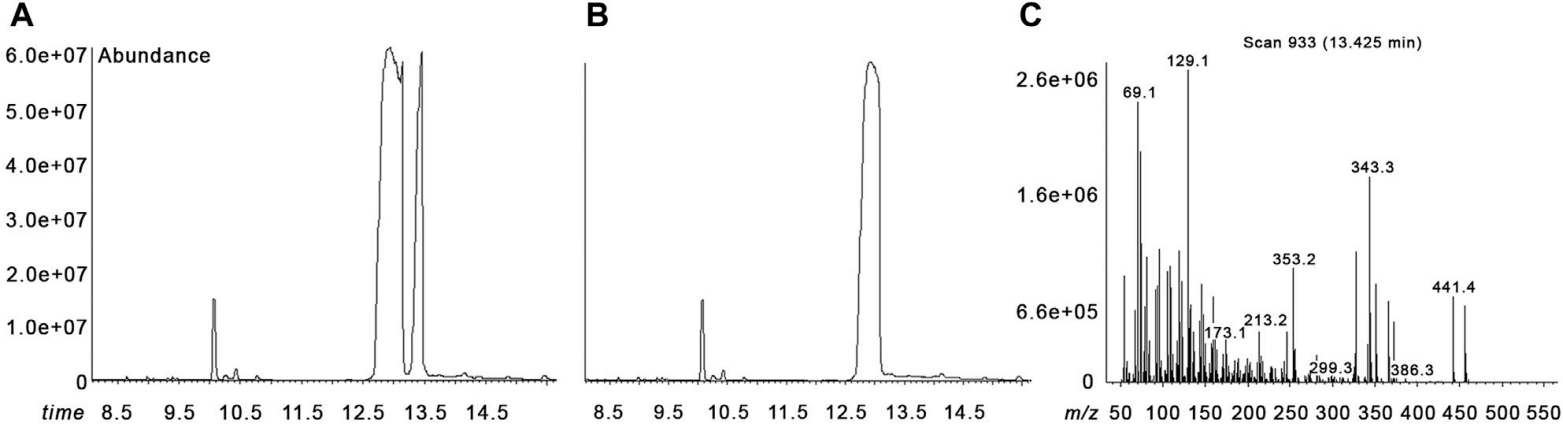
Gas chromatography–mass spectrometry (GC/MS) analysis. Chromatograms of our patient with desmosterolosis **(A)** and of a normal control **(B)**. Mass spectrum of desmosterol **(C).** The three peaks in our patient’s chromatogram are in temporal order from left to right at the time 10.07 min 5-alpha-cholestane (internal standard), at the time 12.96 min cholesterol, and at the time 13.43 min desmosterol **(A)**. In the normal control, the desmosterol peak is not present **(B)**.

Sterol quantitation analysis was performed by gas chromatography–mass spectrometry (GC/MS), which showed a 7-dehydrocholesterol level of 0.2 μMol/L (normal values <7) and a desmosterol level of 1712 μMol/L (normal values <0.1) (Figure 2).

### 3.4 Structural characterization

**FIGURE 3:**
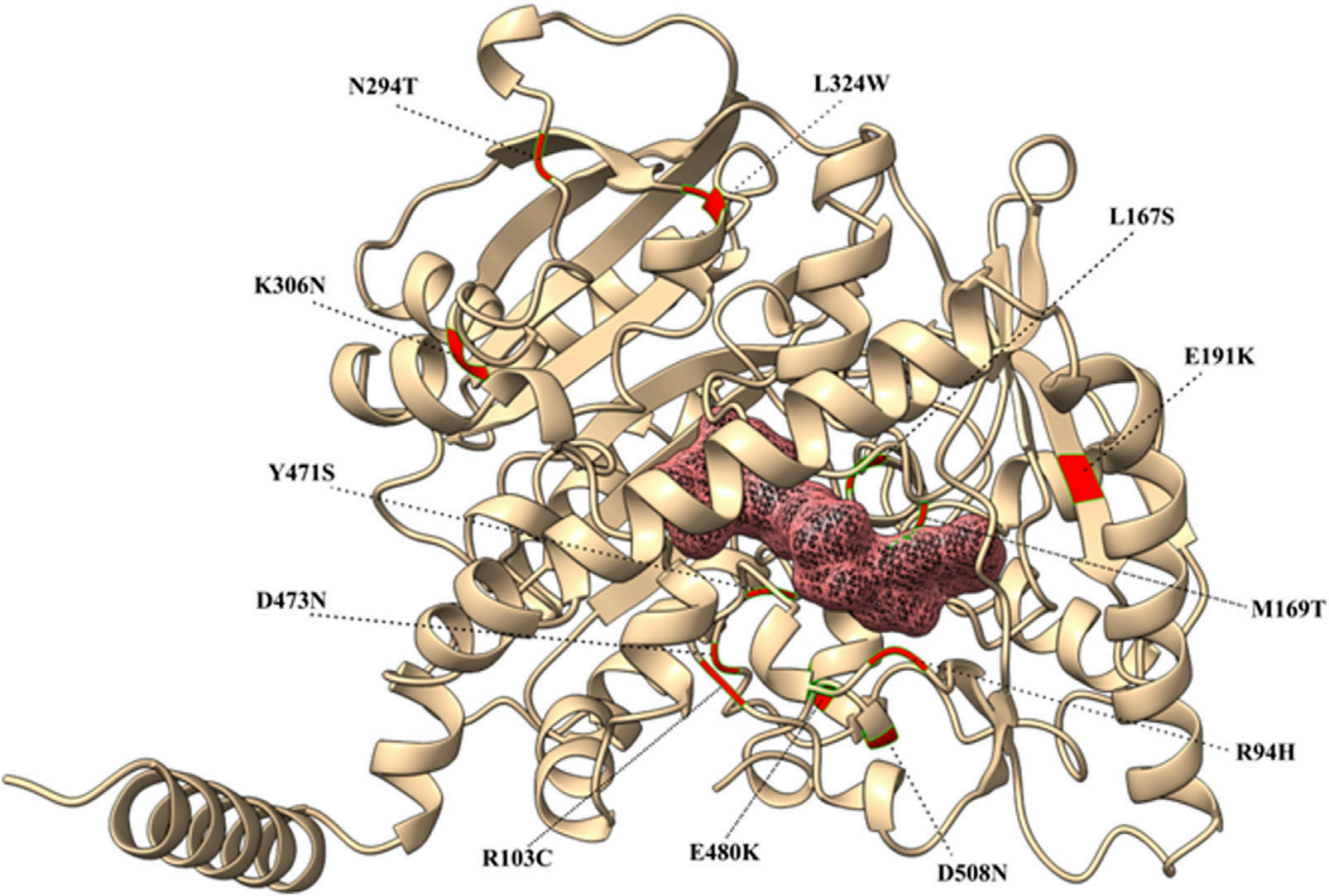
3D structure of the DHCR24 protein with its cofactor (FAD). Missense variants are highlighted in red and labeled accordingly.

Since there is currently no comprehensive evaluation of the structural effects of DHCR24 protein variants, we used AlphaFold to first generate a comprehensive model of the DHCR24 wild-type and then AlphaFill to predict its essential ligand-binding sites. We modeled the proband’s variant, p.M169T, together with all variants known to be related with the same disease, i.e., p.R94H, p.R103C, p.E191K, p.D473N, p.K306N, p.L167S, p.M169T, p.N294T, p.E480K, p.Y471S, and p.L324W, one double mutant, i.e., p.[N294T; K306N], and a low-frequency and putatively benign variant, i.e., p.D508N, as negative control, to comparatively bear out this hypothesis (Figure 3). We observed that, like the proband variant, p.Y471S, p.R94H, and p.L167S fell in the putative FAD-binding site, while p.E191K and p.R103C mapped near the binding site. The remaining variants were scattered in other regions of the protein.

### 3.5 Molecular dynamics simulation

**FIGURE 4:**
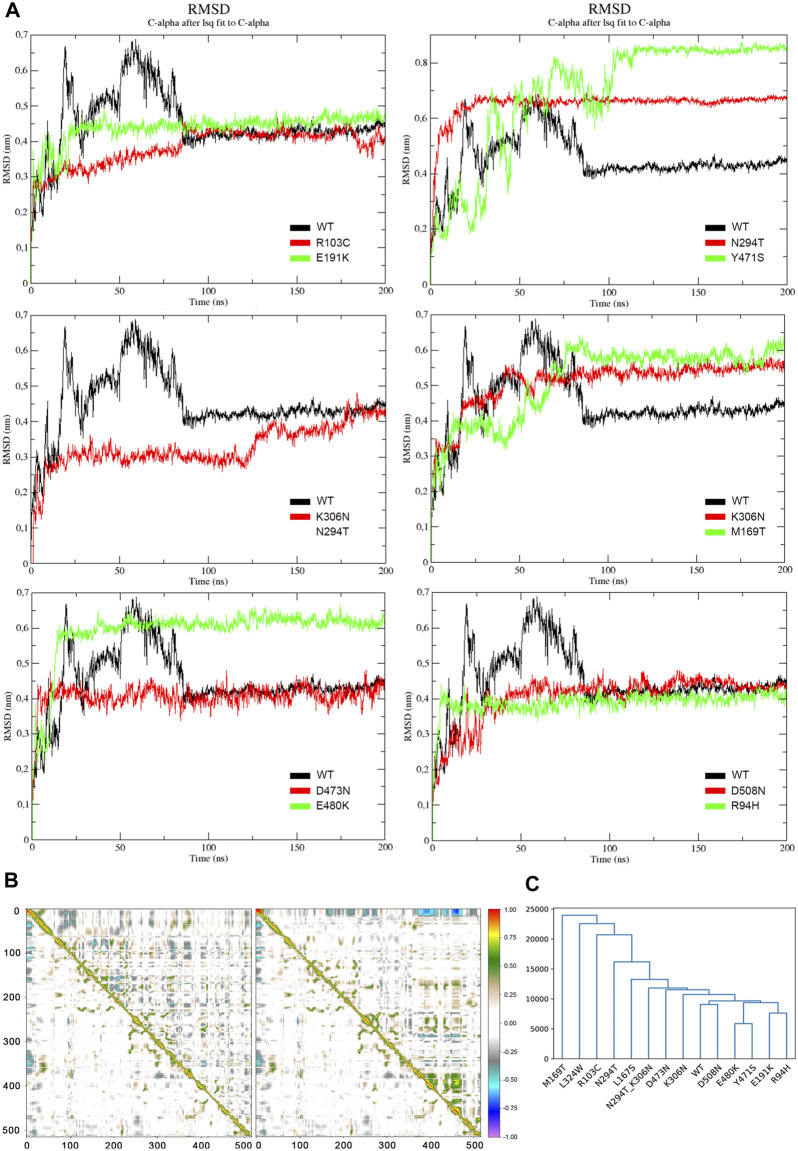
**(A)** RMSD profiles of the heavy atoms of DHCR24. Each panel compares the wild-type (black) with any two different mutants (in red and green). The double mutant p. [N294T; K306N] is represented alone with the wild-type and is colored in red. **(B)**. DCCMs of p.E191K (left) and p.Y471S. In each DCCM, the lower triangular matrix refers to the wild-type protein’s movements and is provided to ease the direct comparison with the mutants’ movements (upper triangles). Perfect correlations between movements of residues are highlighted in red (direct) or violet (inverse). The residues’ positions in the proteins are reported on the *X* and *Y*-axes symmetrically. **(C)** Clustering analysis of the interaction profiles of DHCR24 with FAD throughout the whole simulation time. The adopted similarity metric is the Euclidean distance.

We have simulated all considered DHCR24 protein mutants for 200 simulated *ns* and then estimated the root mean square deviation (RMSD) values of the alpha carbons (C_α_) compared with the wild-type minimized structure to assess their overall protein stability. Overall, we observed that all RMSD profiles reached a plateau after 75 *ns* of simulation, and for this reason, only the remaining 125 *ns* were considered for further analyses. In detail, the wild-type RMSD profile stabilized at ∼0.4 *nm*. Similarly, p.R94H, p.R103C, p.E191K, p.D473N, and the benign variant p.D508N reached the value of ∼0.4 *nm* after a short unstable phase. Conversely, p.M169T, the proband variant, exhibited a higher RMSD profile and stabilized at ∼0.6 *nm* after 90 *ns*. Similarly, another variant falling in the putative FAD-binding site, p.Y471S, reached a stable RMSD profile at approximately 0.8 *nm* after ∼120 *ns*. RMSD values of p.K306N, p.N294T, and p.E480K ranged from 0.5 to 0.6 *nm* but were reached earlier during the simulation. Finally, a lower but more unstable RMSD profile was observed for the double mutant p.[N294T; K306N] protein system (Figure 4A).

The DCCM analysis revealed that the motion of the amino acids of the proband mutant protein did not show any alteration during simulation when compared to the wild-type protein. A similar behavior resulted for all other mutants but p.E191K and p.Y471S. These exhibited an evident alteration of their residues’ movements, which were more general for p.E191K, because of its increased global anticorrelated movements, and more domain-specific for p.Y471S, where we found a strong increase in correlated movements from residues 350 to 450 (Figure 4B).

The effect of the remaining variants on DHCR24 was, therefore, investigated by looking at the transient interactions established with FAD during simulation, compared to the wild-type. In Supplementary Table S2, we report the most important protein–FAD interactions for which we observed an alteration of at least 20% of the interaction frequency in at least one mutant. The proband’s p.M169T mutant presented the most dramatic impact on FAD in terms of the number of dropped interactions. It abolished 50% of the interactions that were instead almost all stably established by the wild-type protein. p.L324W and p.R103C lost 37.5% of the interactions with FAD, while p.N294T lost ∼30% . All the other mutants showed percentages of lost links lower than 30%. It is worth noting that p.Y471S hit an important FAD interaction site and determined the loss of a critical hydrogen bond. It should also be noted that almost all mutants lost the FAD interaction with the p.E174 and p.I510 DHCR24 residues and that the neutral p.D508N mutant maintained all interactions established by the wild-type protein, with only reduced, hence not abolished, interaction frequencies with these residues.

To finally order all mutants by their impact on FAD, we performed a PCA transformation over the first seven principal components, which captured 92.05% of the original total variance, followed by a hierarchical clustering analysis of all observed interactions. The resulting dendrogram (Figure 4C) showed that the “similarity” between wild-type and neutral p.D508N dynamics in the principal component space was higher compared with the other mutants. An interesting case regards p.N294T, which clustered distantly from the wild-type, while p.K306N showed a more similar interaction profile to the wild-type. However, when occurring together, it looks like p.K306N partially compensated the pathogenic impact of p.N294T, thereby conferring a net interaction profile more similar to p.K306N, albeit with the loss of fundamental interactions. Moreover, this analysis confirmed the proband’s variant as the most impacting on the FAD compared to all the others, with those characterized by less FAD interaction drops, i.e., p.E480K, p.Y471S, p.E191K, and p.R94H, having diverse molecular dynamics profiles.

## 4 Discussion

Here, we describe an Italian patient affected by a severe developmental disorder mostly characterized by progressive cerebral and cerebellar atrophy, severe epileptic encephalopathy, spastic quadriplegia, macrocephaly, and visual impairment, harboring the never-described missense variant NM_014762.4:c.506T>C, NP_055577.1:p.M169T in the homozygous state in *DHCR24*, whose locus is phylogenetically conserved through vertebrates and Mammalia. *DHCR24* encodes flavin adenine dinucleotide (FAD)-dependent oxidoreductase, which catalyzes the reduction of the Δ24 double bond of sterol intermediates during cholesterol biosynthesis. Missense variants in this gene have been associated with desmosterolosis, a rare autosomal recessive disorder of cholesterol biosynthesis manifesting a spectrum of severity, from a fatal form observed in a preterm infant to the longest-surviving patient reaching the age of 14 (Dias et al., 2014), and resulting in multiple congenital abnormalities, failure to thrive, and syndromic developmental delay. Additionally, reduced DHCR24 expression occurs in the temporal cortex of AD patients, and overexpression has been observed in adrenal gland cancer cells.

To date, 15 *DHCR24* variants have been associated with desmosterolosis detected in 2 related and 14 unrelated patients. Of them, 11 were either missense variants in homozygosity or compound heterozygosity, and 2 were non-sense variants with either 1 frameshift or 1 splicing variant in the compound heterozygous state; all were classified as pathogenic or likely pathogenic. The missense variant p.M169T discussed in this report has never been described; it is predicted as disease-causing, according to 12 predictor software applications, and its pathogenic role has been confirmed in this study through a biochemical assay that showed high plasma desmosterol levels by gas chromatography–mass spectrometry.

From a structural standpoint, p.M169T had a clear impact on the FAD interaction mechanism (Supplementary Video S1). Such an alteration was also evident in a subset of known *DHCR24* variants associated with desmosterolosis, allowing us to identify at the molecular level the residue interaction network essential for the proper functioning of the protein and its cofactor. p.M169T caused an evident disruption of the entire FAD-binding pocket, while p.E191K triggered a global change in the motions of the protein residues rather than focusing on the cofactor-binding pocket. With different mechanisms, these two turn out to be the most impacting variants on the physiological protein functioning. Additionally, p.Y471S determined the loss of an important hydrogen bond with the FAD, thereby causing a domain-specific strong increase in correlated atomic movements with low FAD interaction modifications.

The association analysis represented in Figure 4C conveys the message that two potential pathogenic mechanisms coexist that may be linked to this set of variants, one affecting the interaction network with FAD and one due to the general or domain-specific derangement of the motions of residues. The former are caused by variants that cluster on the left side of the dendrogram shown in Figure 4C, which are more similar to the proband’s molecular dynamics profile and far from the wild-type, while the latter lie on the right side and have closer profiles to the wild-type in terms of broken or reduced FAD interactions. Moreover, it is also interesting to note that almost all mutants lost the FAD interactions through the p.E174 and p.I510 DHCR24 residues and that the neutral p.D508N mutant maintained all interactions established by the wild-type protein, with only reduced, hence not abolished, interactions with these residues (Supplementary Table S3). Therefore, a third aspect that may be considered in evaluating the impact of future *DHCR24* variants is related to whether they hit these two residues directly or indirectly. Concerning instead the double mutant p.[N294T; K306N], we verified that, when considered separately, the p.N294T mutant appeared to be more different from the wild-type than the p.K306N variant. However, when these two variants occurred together, p.K306N seemed to partly compensate for the harmful effects of p.N294T, resulting in an overall interaction profile that is more similar to p.K306N, albeit with the loss of some fundamental interactions. This fourth aspect suggests that variants that moderately impact the FAD interaction network may occur together to globally modulate, by exacerbating or compensating, their individual pathogenic effects, thereby explaining the phenotypic diversity of patients.

Prior studies have noted a low genotype–phenotype correlation in desmosterolosis (Schaaf et al., 2011; Dias et al., 2014), which can be considered in the differential diagnosis of patients who present with concurrent defects of cholesterol synthesis (reported for 46%, i.e., 6 out of 13 subjects with pathogenic *DHCR24* variants and available clinical information), cerebral malformations, in particular micro/macrocephaly (69%) and agenesis of the corpus callosum (53%), facial dysmorphisms, in particular retromicrognathia (53%), cleft palate (38%), limb anomalies (46%), delay in growth and development (61%), and visual impairments (38%). This patient expands the clinical spectrum by adding the features of severe epileptic encephalopathy and reduced basal ganglia, thalamus, and spinal cord volume. All clinical features of the considered patients are reported in Supplementary Table S3.

Regarding genotypes, 11 missense variants (5 in or near the FAD-binding pocket) and 1 frameshift, 1 splicing, and 2 nonsense variants are reported in Supplementary Table S3. Non-sense variants, p.R494* and p.Q402*, were found to be in compound heterozygosity with a frameshift, p.F415VfsTer11, and splicing, c.1218+1G>A, variant. Although we could not obtain any clinical description for the patient carrying p.[R494*];[F415VfsTer11], the one carrying p.[Q402*];[c.1218+1G>A] exhibited most traits common to other patients, except a skin edema. A total of 5 missense variants, all in compound heterozygosity, were located far from the FAD-binding domains. The remaining 6 were within or near the FAD-binding pocket; half of them were in homozygosity. Overall, 5 of these 6 missense variants correlated biochemically with high desmosterol levels and were the same variants, i.e., p.M169T, p.R103C, p.Y471S, p.E191K, and p.R94H, which lie at the extremities of the dendrogram shown in Figure 4C, since they cause the worst structural impact to the DHCR24 protein.

Considering the significantly variable clinical expressivity observed in all patients reported in Supplementary Table S3, we have observed that macrocephaly was reported for patients carrying the most structurally severe DHCR24 variants, i.e., p.M169T, p.Y471S, and p.[R94H];[E480K]. Microcephaly, on the contrary, occurred in three patients carrying p.E191K and one p.R103C, which are spatially close but not within the FAD-binding pocket, thereby causing milder structural consequences. Interestingly, these two conditions do not share any *DHCR24* variant. Cleft palate and limb anomalies were not reported for patients carrying neither p.M168T nor R103C. All the other traits were not strikingly associated with any other variant.

In conclusion, this report presents a novel *DHCR24* missense variant, p.M169T, shows its molecular mechanisms, and links them to the desmosterolosis presentation. To do that, we first measured the proband’s sterol quantity by gas chromatography–mass spectrometry and then studied the protein structural consequences of p.M169T alone and compared with all other existing variants in this gene by means of molecular dynamics simulation. Then, we found three classes of pathogenic mechanisms that should be considered in the assessment of future *DHCR24* variants: (i) perturbation of the FAD cofactor interaction network, (ii) global or domain-specific derangement of residue motions with or without a reduced perturbation of the FAD interaction network, and (iii) a direct or indirect involvement/alteration of p.E174 and p.I510 DHCR24 residues. Finally, we observed some genotype–phenotype associations, particularly regarding cerebral, facial, and limb malformations observed in the patients considered in this study. However, due to the current limited number of known *DHCR24* variants and the multi-trait nature of the disease, it is required to confirm these assessments when new variants emerge.

## Data Availability

The original contributions presented in the study are publicly available. These data can be found online at: https://www.ebi.ac.uk/ena/browser/view/PRJEB70403.

## Supplementary material

SUPPLEMENTARY TABLE S1 Pathogenicity prediction scores of 14 software packages. When available, a functional categorical assessment is additionally provided.

SUPPLEMENTARY TABLE S2 The most important interactions between the DHCR24 residues (columns) and the FAD cofactor for each mutant (rows). Cells contain “frequency” values, meant as the percentage of time each interaction occurred over the entire simulation time. Values are colored red (high frequencies) and yellow (low frequencies).

SUPPLEMENTARY TABLE S3 Literature review of *DHCR24* variants associated with desmosterolosis.

SUPPLEMENTARY VIDEO S1 Molecular dynamics simulation showing the DHCR24 p.M169T impact on the FAD interaction.
